# Fogs and Gas-Poisoning

**Published:** 1902-10-25

**Authors:** 


### FOGS AND GAS-POISONING.

With the season approaching in which fogs are apt to play so large a part in diminishing the pleasures of town life it becomes a question of considerable interest to inquire how far the obvious and acknowledged evils of a town fog are not added to by an unsuspected form of poisoning due to the highly noxious gases which at such times are largely employed by the gas companies for the adulteration of the honest coal gas with which they are imagined to supply their consumers. Coal gas contains a certain proportion of carbon monoxide—6 to 8 per cent.—and it is principally to the presence of this comparatively small amount of carbon monoxide that it owes such poisonous properties as it possesses; but water-gas contains 40 per cent. of this compound, and is thus a very much more poisonous affair. Yet from the companies' point of view water-gas possesses several great advantages. First of all it is cheap. But what is even more important is that it can be quickly produced in large quantities as occasion may require, whereas the production of ordinary gas must be carried on continuously, involving the use of enormous gasometers so as to meet any extra demand. Hence it is that when the onset of foggy weather suddenly doubles the demand for gas the difficulty can be, and it is believed is, met by largely increasing the proportion of water-gas contained in the product supplied to the consumers. Now this would not matter if all the gas-fittings were as they should be. Water-gas is a very good gas; for heating purposes it is admirable, and for incandescent burners it is, we believe, even better than the tarry, smoky, so-called illuminating gas. But it is a very deadly poison, and unfortunately our fittings are not all as they should be, and from this arise many troubles and much danger. Naturally the gas companies will say that we should look after our fittings. In this there is much reason, and would be more were it not so obvious that the gas companies themselves are amongst the greatest sinners in regard to leaky pipes. The amount of gas which the companies allow to escape into the soil is something almost incredible. Of late years the gas soaked condition of the soil has been painfully demonstrated by the explosions which occur from time to time in the electric light boxes; but if gas can leak into an electric light box so it can into the drains, cellars and ground floors of adjacent houses, and when this gas contains a large proportion of poisonous carbon monoxide the results have not infrequently been fatal. Yet even although the consequences of breathing this gas may not prove fatal, they may be very unpleasant and injurious to health. Moreover, there is an uncomfortable suspicion that iron pipes which do very well for ordinary gas are more or less permeable to carbon monoxide. Hence on every hand, whether we regard the defective fittings or the gas-soaked soil any large increase in the most, if not the only, poisonous constituent of coal-gas, is a matter to be regarded with considerable anxiety. We cannot afford to overlook the statistics which have lately come from America where water-gas is far more extensively used than it is here. The secretary of the Massachusetts State Board of Health lately wrote to *Public Health* saying that in the 13 years previous to the introduction of water-gas in Massachusetts the number of deaths registered as due to poisoning from illuminating gas was eight; while in the 13 years following the introduction of water-gas the number of deaths registered as due to illuminating gas was 459. Again, in regard to the city of Boston, the number of deaths recorded as due to poisoning from illuminating gas gradually increased from 14 in the year 1892 to 58 in 1898, and 51 in 1899, dropping in 1900 and 1901 to 25 and 14 respectively, the explanation being that "the percentage of water-gas in the supply furnished to that city gradually increased to about 95 per cent. in 1898 and 1899, and then gradually diminished to less than 50 per cent. in the remaining years." This reduction, we believe, took place in consequence of the strong protests which the growing frequency of deaths from gas poisoning had given rise to. Under these circumstances, and in view of the known tendency of the gas companies to resort to water gas when large demands are suddenly made upon their resources by the onset of fogs, we think it would be well for medical men to be on the look-out for fog dangers in the form of gas-poisoning, as well as in the more generally recognised form of bronchitis and other respiratory diseases. As is well known, even ordinary gas, and in quite small proportions, acts deleteriously if breathed for long, producing headache and general depression. A very slight escape, so slight as not to be recognised as gas at all, but merely to produce a sense of stuffiness, will cause relaxed sore throat; indeed, there is reason to believe that many so-called "drain smells" and "drain throats" are merely the result of undiscovered gas leaks. But when the gas consists largely of carbon monoxide the effects are much more serious. The early symptoms of poisoning by carbon monoxide, so far as the patient's feelings are concerned, are giddiness and headache, with a sense of heaviness, heat in the skin of the face, the chief site of the headache being across the fore-head, and both the headache and the giddiness increasing as the exposure to the cause continues. Associated with these there is generally a subjective feeling of strong pulsation in the temporal arteries. Then there are noises in the ears, and sometimes a sense of intense anxiety and oppression about the heart with nausea and even vomiting; sometimes a sense of excitement and exaltation, almost allied to that produced by alcohol; while in the further stages a sense of helplessness and indifference comes on, which leads the patient to yield to the drowsiness rather than to fly from it. Returning, however, to the milder symptoms—the burning face, the frontal headache, the throbbing arteries, the noises in the ears—when these are found to recur or to increase with the onset of foggy weather, let us look out for gas, and see whether they do not mean that some small leak which with good gas had caused but little trouble, is giving rise to mischief now that the gas consists largely of the poisonous carbon monoxide.

